# Therapeutic potential of the mitochondria-targeted antioxidant MitoQ in mitochondrial-ROS induced sensorineural hearing loss caused by *Idh2* deficiency

**DOI:** 10.1016/j.redox.2018.11.013

**Published:** 2018-11-20

**Authors:** Ye-Ri Kim, Jeong-In Baek, Sung Hwan Kim, Min-A Kim, Byeonghyeon Lee, Nari Ryu, Kyung-Hee Kim, Deok-Gyun Choi, Hye-Min Kim, Michael P. Murphy, Greg Macpherson, Yeon-Sik Choo, Jinwoong Bok, Kyu-Yup Lee, Jeen-Woo Park, Un-Kyung Kim

**Affiliations:** a Department of Biology, College of Natural Sciences, Kyungpook National University, Daegu, Republic of Korea; b School of Life Sciences, KNU Creative BioResearch Group (BK21 Plus Project), Kyungpook National University, Daegu, Republic of Korea; c Department of Aroma-Applied Industry, College of Herbal Bio-industry, Daegu Haany University, Gyeongsan, Republic of Korea; d Medical Research Council (MRC)-Mitochondrial Biology Unit, University of Cambridge, Cambridge CB2 0XY, United Kingdom; e Antipodean Pharmaceuticals Inc, L2 14 Viaduct Harbour Rd, Auckland, New Zealand; f Department of Anatomy, Yonsei University College of Medicine, Seoul, Republic of Korea; g BK21PLUS Project for Medical Science, Yonsei University College of Medicine, Seoul, Republic of Korea; h Department of Otorhinolaryngology, Yonsei University College of Medicine, Seoul, Republic of Korea; i Department of Otorhinolaryngology-Head and Neck Surgery, School of Medicine, Kyungpook National University, Daegu, Republic of Korea; j Department of Biochemistry, School of Life Sciences and Biotechnology, College of Natural Sciences, Kyungpook National University, Daegu 41566, Republic of Korea

**Keywords:** Idh2, NADP^+^, ROS, Hearing loss, Antioxidant, MitoQ

## Abstract

Mitochondrial NADP^+^-dependent isocitrate dehydrogenase 2 (IDH2) is a major NADPH-producing enzyme which is essential for maintaining the mitochondrial redox balance in cells. We sought to determine whether IDH2 deficiency induces mitochondrial dysfunction and modulates auditory function, and investigated the protective potential of an antioxidant agent against reactive oxygen species (ROS)-induced cochlear damage in *Idh2* knockout (*Idh2*^*−/−*^) mice. *Idh2* deficiency leads to damages to hair cells and spiral ganglion neurons (SGNs) in the cochlea and ultimately to apoptotic cell death and progressive sensorineural hearing loss in *Idh2*^*−/−*^ mice. Loss of IDH2 activity led to decreased levels of NADPH and glutathione causing abnormal ROS accumulation and oxidative damage, which might trigger apoptosis signal in hair cells and SGNs in *Idh2*^*−/−*^ mice. We performed *ex vivo* experiments to determine whether administration of mitochondria-targeted antioxidants might protect or induce recovery of cells from ROS-induced apoptosis in *Idh2*-deficient mouse cochlea. MitoQ almost completely neutralized the H_2_O_2_-induced ototoxicity, as the survival rate of *Idh2*^*−/−*^ hair cells were restored to normal levels. In addition, the lack of IDH2 led to the accumulation of mitochondrial ROS and the depolarization of *ΔΨ*_*m*_, resulting in hair cell loss. In the present study, we identified that IDH2 is indispensable for the functional maintenance and survival of hair cells and SGNs. Moreover, the hair cell degeneration caused by IDH2 deficiency can be prevented by MitoQ, which suggests that *Idh2*^*−/−*^ mice could be a valuable animal model for evaluating the therapeutic effects of various antioxidant candidates to overcome ROS-induced hearing loss.

## 1 Introduction

The balance between reactive oxygen species (ROS) production and antioxidant defenses is essential for maintaining cellular homeostasis. Under physiologic conditions, cells maintain redox balance through the generation and elimination of ROS [1]. Mitochondria are a major endogenous source of cellular ROS, where O_2_^−^ is generated by electron leakage from complex I to IV of the electron-transport chain [2], [3]. Various enzymatic (superoxide dismutase (SOD) or glutathione peroxidase (GPx)) and nonenzymatic (vitamin C or E) antioxidant systems contribute to eliminating ROS and maintaining redox homeostasis. Excessive ROS constantly attack lipids, proteins, and DNA in cells, leading to severe and irreversible oxidative damage. Several ROS-related studies have demonstrated that increased oxidative damage plays a crucial role in a variety of pathologic conditions, including cancer, neurodegenerative diseases, and aging [4], [5].

Hearing loss, a common sensory disorder that is caused by different genetic or environmental factors, is also triggered by the accumulation of continuous oxidative stress with age [6]. Ototoxic drugs, noise exposure, and aging are common environmental factors that disrupt intracellular redox balance [6], [7], [8], and pathogenic mutations in the genes that are involved in the redox system are the major genetic causes of ROS-induced hearing loss. For example, methionine sulfoxide reductase B3 (MsrB3) that specifically reduces methionine-R-sulfoxide of proteins to methionine, is the causative gene of autosomal recessive non-syndromic hearing loss DFNB74 [9]. Because methionine residues in proteins are a major target of oxidization to methionine sulfoxide by ROS, this enzyme is responsible for preserving the biological activity of proteins after oxidative damage due to ROS [10], [11]. In addition, since cochlear cells are highly sensitive to disturbances in energy metabolism, mitochondrial decay may particularly affect the cochlea. Mutations in mitochondrial DNA (mtDNA) or in several nuclear genes coding mitochondrial proteins have been known to be associated with progressive hearing loss [12]. Major mtDNA mutations occur in the genes encoding mitochondrial oxidative phosphorylation complexes and lead to mitochondrial dysfunction. Hyperaccumulation of mitochondrial ROS decreased mitochondrial membrane potential, and activated apoptotic pathways, eventually causing hair cell death.

Mitochondrial NADP^+^-dependent isocitrate dehydrogenase 2 (IDH2) catalyzes the oxidative decarboxylation of isocitrate to α-ketoglutarate, synthesizing NADPH. In mitochondria, the redox balance is mainly determined by the ratios of several redox couples, such as reduced glutathione (GSH)/oxidized glutathione (GSSG) and thioredoxin_red_/thioredoxin_oxid_. The GSH/GSSG couple has been considered the primary determinant of the intracellular redox state, and the GSH/GSSG ratio is regulated by antioxidant enzymatic scavengers, including glutathione reductase (GR) and GP_X_ [13], [14]. Given the role of NADPH as the electron donor for GR-mediated GSH generation, IDH2, which increases NADPH levels by converting NADP^+^ to NADPH, has been highlighted as a major contributor to the antioxidant defense system in various tissues and cells [15], [16], [17], [18], [19], [20], [21]. It has been shown that IDH2 protects organs against various diseases, including cancer, skin pigmentation [22] and renal dysfunction [23]. In addition, endothelium-dependent vasorelaxation is impaired, and the concentration of bioavailable NO is decreased in the aortic ring in *Idh2* knockout (*Idh2*^*−/−*^) mice [24].

However, the physiological association between *Idh2* and auditory function and its underlying mechanisms are not fully understood. Therefore, we sought to determine whether *Idh2* deficiency induces mitochondrial dysfunction and modulates auditory function, and investigated the protective potential of an antioxidant agent against ROS-induced cochlear damage in *Idh2*^*−/−*^ mice.

## 2 Materials and methods

### 2.1 Animals

*Idh2*^*−/−*^ mice were bred [19], and mice of their background strain (C57BL/6N) were used as a wild-type (*Idh2*^*+/+*^) control. For genetic identification of the *Idh2*^*−/−*^ mice, tail DNA genotyping was performed. Mice were allowed free access to water and standard mouse chow. Temperature (23 ± 2 °C), humidity (50 ± 5%) and a daily 12 h light–dark cycle were maintained in the Central Laboratory Animal Facility of Kyungpook National University. All animal procedures were conducted in accordance with the Institutional Animal Care guidelines issued by the Committee of Animal Research at Kyungpook National University (2017–0104).

### 2.2 Reverse-transcription polymerase chain reaction (RT-PCR)

RNA was extracted from the inner ear of mice using an RNeasy® Mini Kit (Qiagen, Hilden, Germany) in accordance with the manufacturer's instructions. RNA was reverse transcribed to cDNA using a High-Capacity cDNA Reverse Transcription Kit (Applied Biosystems, Foster City, CA, USA) according to the manufacturer's protocol. cDNAs were PCR-amplified. The *glyceraldehyde 3-phosphate dehydrogenase* (*Gapdh*) gene was used as an internal control.

### 2.3 Western blot analysis

The whole-cell lysates or tissue homogenates (20 µg) prepared from cochlear explants was used for western blotting. The proteins were detected using the appropriate primary and secondary antibodies. After a series of washes, membranes were developed using an enhanced chemiluminescent detection system. Values are normalized to β-actin (loading control). The primary antibodies used in this study were as follows: rabbit polyclonal anti-IDH2 (1:1000; Novus Biologicals, Littleton, CO, USA), rabbit polyclonal anti-OXPHOS complex subunits: NDUFA9 (1:500; Thermo Fisher Scientific, Rockford, IL, USA), SDHA (1:500; Thermo Fisher Scientific, Rockford, IL, USA), UQCRC2 (1:5000; Thermo Fisher Scientific, Rockford, IL, USA), COX4 (1:500; Thermo Fisher Scientific, Rockford, IL, USA) and ATP5A1 (1:1000; Thermo Fisher Scientific, Rockford, IL, USA) and rabbit polyclonal anti-β actin (1:2000; Cell Signaling Technology, Danvers, MA, USA). Goat anti-rabbit-lgG-HRP was used as the secondary antibody (1:2000; Cell Signaling Technology, Danvers, MA, USA).

### 2.4 Auditory brainstem response (ABR) measurement

Mouse auditory function was assessed by measuring ABR with an ABR workstation-System 3 (Tucker Davis Technology, Alachua, FL, USA), as previously described [25]. Auditory function was measured with click stimuli and tone burst sound frequencies of 8, 16, and 32 kHz, and acoustic thresholds of sound pressure level (SPL) were determined using BioSigRP software (Tucker Davis Technology, Alachua, FL, USA).

### 2.5 Immunofluorescence and histological analysis

Inner ears were harvested and fixed with 4% paraformaldehyde (PFA) in phosphate-buffered saline (PBS). The fixed inner ear tissues were embedded in paraffin. Paraffin-embedded tissues were serially sectioned at 7 µm and then subjected to immunofluorescence analysis or hematoxylin-eosin (H&E) staining. The paraffin sections were incubated for 1 h at 65 °C, deparaffinized with xylene, and rehydrated using a graded ethanol series. The tissue sections were permeabilized with 0.1% Triton X-100 in 1X PBS (PBS-Tx) for 30 min and blocked using a blocking solution containing 5% normal goat serum (NGS) in PBS-Tx for 1 h at room temperature (RT). The sections were then incubated overnight at 4 °C with rabbit anti-NADPH (Biorbyt, Cambridge, UK) and mouse anti-COX4 (Abcam, Cambridge, UK). After washing with PBS, the tissue sections were incubated for 1 h at RT with secondary antibodies. The secondary antibodies used for immunofluorescence were Alexa Fluor 488-conjugated goat anti-mouse IgG and 555-conjugated goat anti-rabbit IgG (Thermo Fisher Scientific, Rockford, IL, USA). 4′-6-Diamidino-2-phenylindole (DAPI, 1 µg/mL) was used to visualize nuclei.

### 2.6 Terminal deoxynucleotidyl transferase dUTP nick end labeling (TUNEL) assay

To assess apoptotic cell death in the inner ear, we evaluated DNA fragmentation using the TUNEL assay according to the manufacturer's protocol (Roche Biochemicals, Mannheim, Germany). Paraffin-embedded inner ear sections were deparaffinized and rehydrated. They were then permeabilized with PBS-Tx and 0.1% sodium citrate in distilled water for 20 min at RT and stained with TUNEL working solution for 1 h at 37 °C, without light. The specimens were mounted on glass slides using fluoromount (Sigma-Aldrich, Saint Louis, MO, USA) and visualized using a Zeiss Axio Imager A2 fluorescence microscope (Carl Zeiss, Oberkochen, Germany).

### 2.7 Enzyme assays

Supernatants from homogenized mitochondrial pellets were each added to 1 mL of 40 mM tris buffer (pH 7.4) containing NADP^+^ (2 mM), MgCl_2_ (2 mM), and isocitrate (5 mM). IDH2 activity was measured by monitoring NADPH production at 340 nm at 25 °C. One unit of IDH2 activity was defined as the amount of enzyme needed to catalyze the production of 1 mmol of NADPH per minute. Total GSH levels and the GSH/GSSG ratio were measured using a commercially available GSH/GSSG Ration Detection Assay Kit (Abcam, Cambridge, UK) according to the manufacturer's instructions.

### 2.8 Culture and histological evaluation of mouse cochlear explants

Primary cochlear explants were prepared from postnatal day (P) 3 mice. The dissected organs of Corti were incubated with culture medium composed of high-glucose Dulbecco's modified Eagle's medium (DMEM; HyClone, Logan, UT, USA) containing 10% fetal bovine serum (FBS; HyClone, Logan, UT, USA) and ampicillin (10 μg/mL) in a humidified atmosphere of 5% CO_2_ at 37 °C. After 16 h incubation, organs of Corti were treated with 500 nM MitoQ in dimethyl sulfoxide (DMSO) or 500 nM decyl triphenylphosphonium (dTPP) in DMSO provided by Prof. Michael P. Murphy. At the same time, some groups were treated with 5 μM rotenone (Sigma-Aldrich, Saint Louis, MO, USA) or 10 μM antimycin A (Sigma-Aldrich, Saint Louis, MO, USA) diluted in culture medium for 1 h. After 1 h incubation, 0.05 mM hydrogen peroxide (H_2_O_2_) was added.

### 2.9 Histological evaluation of mouse cochlear explants

At the end of the 5-day incubation period, all cochlear explants were washed with PBS, fixed with 4% PFA in PBS for 15 min, and permeabilized for 30 min at RT. Permeabilized samples were blocked with 5% NGS diluted in PBS-Tx for 1 h at RT and then stained with Alexa Fluor® 488 or 555-conjugated phalloidin (1:1000; Invitrogen, Eugene, OR, USA) in PBS-Tx for 3 h at RT. The specimens were rinsed three times with PBS and mounted on glass slides using fluoromount (Sigma-Aldrich, Saint Louis, MO, USA). For immunohistochemical quantification, the inner hair cells (IHCs) and outer hair cells (OHCs) were separately counted along the 6 regions from the middle of each cochlear explant. Perfectly shaped hair cells in each region, with a length of 200 µm of basilar membrane, were counted. Each experiment was performed independently and repeated at least three times.

### 2.10 Determination of mitochondrial ROS levels

MitoSOX-red (Molecular Probes, Eugene, OR, USA) is a fluorogenic indicator of superoxide generated specifically from mitochondria [26]. At the end of the 3-day incubation period, all cochlear explants were washed with PBS and stained with 5 μM MitoSOX-red for 10 min in a humidified atmosphere of 5% CO_2_ at 37 °C. After washing with PBS, the specimens were visualized using a Zeiss Axio Imager A2 fluorescence microscope (Carl Zeiss, Oberkochen, Germany).

### 2.11 Determination of mitochondrial membrane potential (*ΔY*_*m*_)

Mitochondrial membrane potential was estimated using the cationic fluorescent dye MitoProbe™ JC-1 (Invitrogen, Eugene, OR, USA) according to the manufacturer's instructions. At the end of the 3 or 5-day incubation period, all cochlear explants were washed with PBS and incubated with 2 μM JC-1 for 50 min in the dark. To confirm the sensitivity of JC-1, the cochlear explants were not treated with any drugs, 50 μM carbonyl cyanide 3-chlorophenylhydrazone (CCCP) was added before treatment with JC-1, and the samples were incubated. If the cells have a normal range of mitochondrial membrane potentials, fluorescence emission shift from green to red.

### 2.12 Statistical analyses

Statistical analyses were performed using 2-tailed Student's *t*-tests; *P* < 0.05 was considered statistically significant. The data were analyzed by comparing treated and untreated contralateral structures or by comparing treated and control mice.

## 3 Results

### 3.1 Loss of *Idh2* leads to progressive sensorineural hearing loss in mice

Because inner ear expression of the *Idh2* gene in the *Idh2*^*−/−*^ mice [27] has not been confirmed in previous studies, we first examined *Idh2* expression in the inner ear and confirmed that *Idh2* mRNA and protein expression was completely absent in the inner ear of *Idh2*^*−/−*^ mice, while it was abundantly expressed in *Idh2*^*+/+*^ mice (Supplementary Fig. 1). The changes in the hearing threshold of *Idh2*^*+/+*^ and *Idh2*^*−/−*^ mice were then followed-up for 12 months by ABR tests with a click stimulus and frequency-specific stimuli at 8, 16, and 32 kHz to investigate whether lack of *Idh2* affects normal hearing function (Fig. 1). No significant differences were found between *Idh2*^*+/+*^ and *Idh2*^*−/−*^ mice until 2 months after birth. However, after 3 months of age, the hearing ability of the *Idh2*^*−/−*^ mice began to deteriorate significantly compared with the hearing ability of *Idh2*^*+/+*^ mice, eventually resulting in profound hearing loss after 10 months of age. The ABR threshold gap between the *Idh2*^*+/+*^ and *Idh2*^*−/−*^ mice gradually increased at all frequencies (Supplementary Fig. 2), which indicates that *Idh2* deficiency leads to the continuous accumulation of hearing damage with age. Moreover, even if this pattern of hearing loss was consistent at all tested frequencies, the progression of hearing loss was more rapid at mid (16 kHz) and high (32 kHz) frequencies than at low (8 kHz) frequencies (Supplementary Fig. 2). This result indicates that *Idh2* deficiency leads to progressive sensorineural hearing loss in mice, suggesting an important role of *Idh2* in the auditory pathway.

**Fig. 1:**
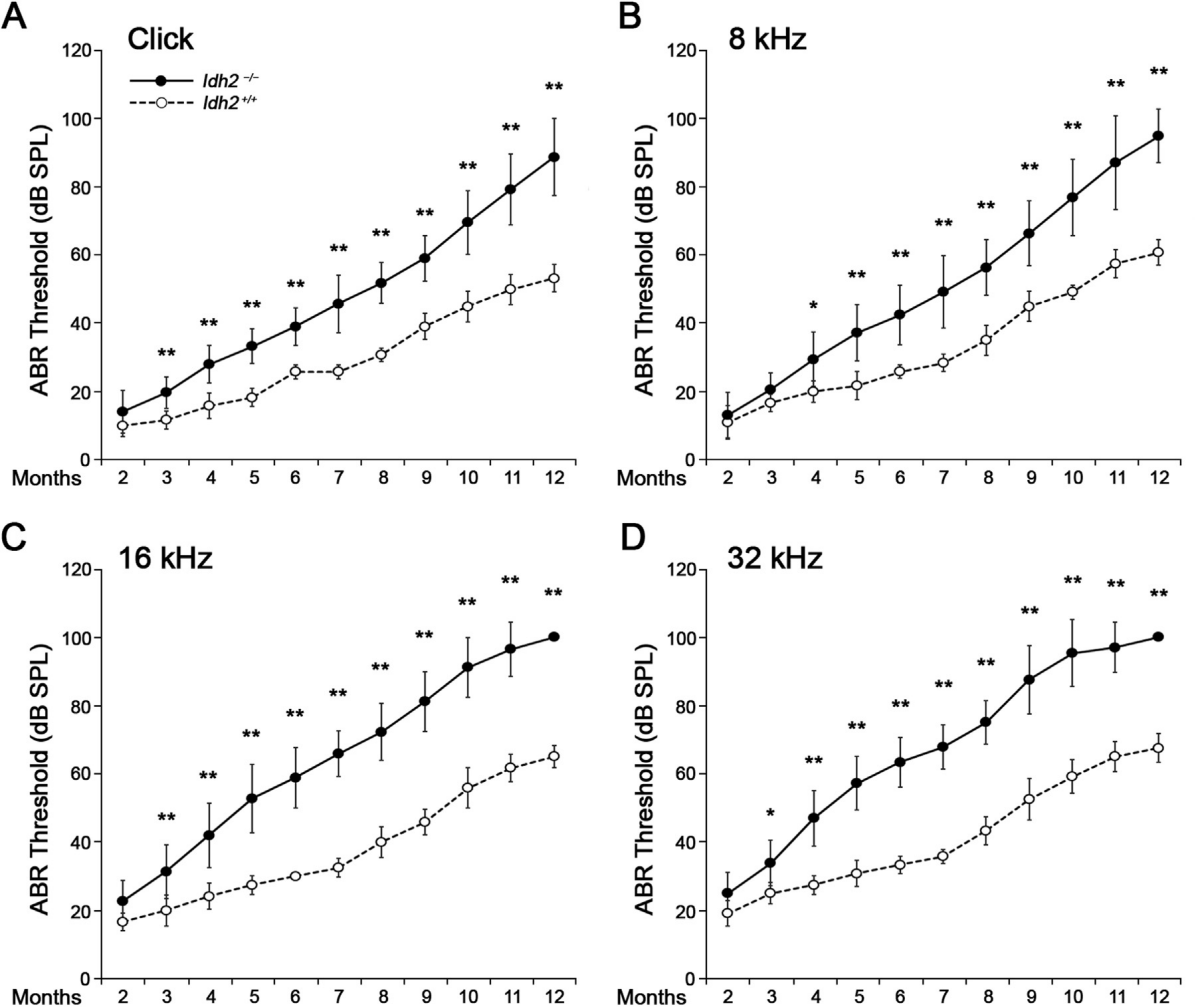
**ABR hearing thresholds of the*****Idh2***^***+/+***^**and*****Idh2***^***−/−***^**mice as a function of age.** The changes in the hearing threshold of *Idh2*^*+/+*^ (white circle with dotted line, *n* = 6) and *Idh2*^*−/−*^ (black circle with solid line, *n* = 18) mice were represented using line graphs. The ABR thresholds were measured for 12 months with a click (A) and tone burst (8, 16, and 32 kHz) (B, C and D) stimuli. Data are shown as the means ± SEM. *p < 0.05, **p < 0.005.

### 3.2 IDH2 deficiency causes damage to hair cells and spiral ganglion neurons (SGNs), leading to apoptosis

To investigate the immediate cause of hearing loss in *Idh2*^*−/−*^ mice, we first examined the histological features of cochlear sections from *Idh2*^*+/+*^ and *Idh2*^*−/−*^ mice through H&E staining at 2 months of age when there was no difference in ABR threshold between *Idh2*^*+/+*^ and *Idh2*^*−/−*^ mice and at 10 months of age when *Idh2*^*−/−*^ mice showed profound hearing loss at 16 and 32 kHz. In the cochlea of 2-month-old mice, no distinguishable differences were detected between *Idh2*^*+/+*^ and *Idh2*^*−/−*^ mice (Supplementary Fig. 3). In contrast, at 10 months of age, obvious damage was observed in the organ of Corti and in the SGNs in *Idh2*^*−/−*^ cochlea (Fig. 2A). While both the inner and outer hair cells from 10-month-old *Idh2*^*+/+*^ cochlea were intact, *Idh2*^*−/−*^ cochlea had morphological degeneration of the hair cells (Fig. 2A d-f, d′-f′). Moreover, the most noticeable difference was found in the spiral ganglion, which showed evident loss of SGNs in the *Idh2*^*−/−*^ cochlea (Fig. 2A g-i, g′-i′). Quantitative analysis confirmed that the loss of SGNs significantly differed from apical to basal cochlear turns, whereas there were no significant differences in stria vascularis thickness and spiral ligament area between *Idh2*^*+/+*^ and *Idh2*^*−/−*^ cochlea (Fig. 2B). Importantly, all these damages were more severe in the basal turn than in the apical turn, which was consistent with the mid- and high-frequency-dominant progression of hearing loss observed in the ABR test of *Idh2*^*−/−*^ mice. A highly increased number of TUNEL-positive cells in the organ of Corti and the SGNs of *Idh2*^*−/−*^ cochlea suggested that *Idh2* deficiency causes apoptosis of hair cells and SGNs, eventually leading to hearing loss (Fig. 3).

**Fig. 2:**
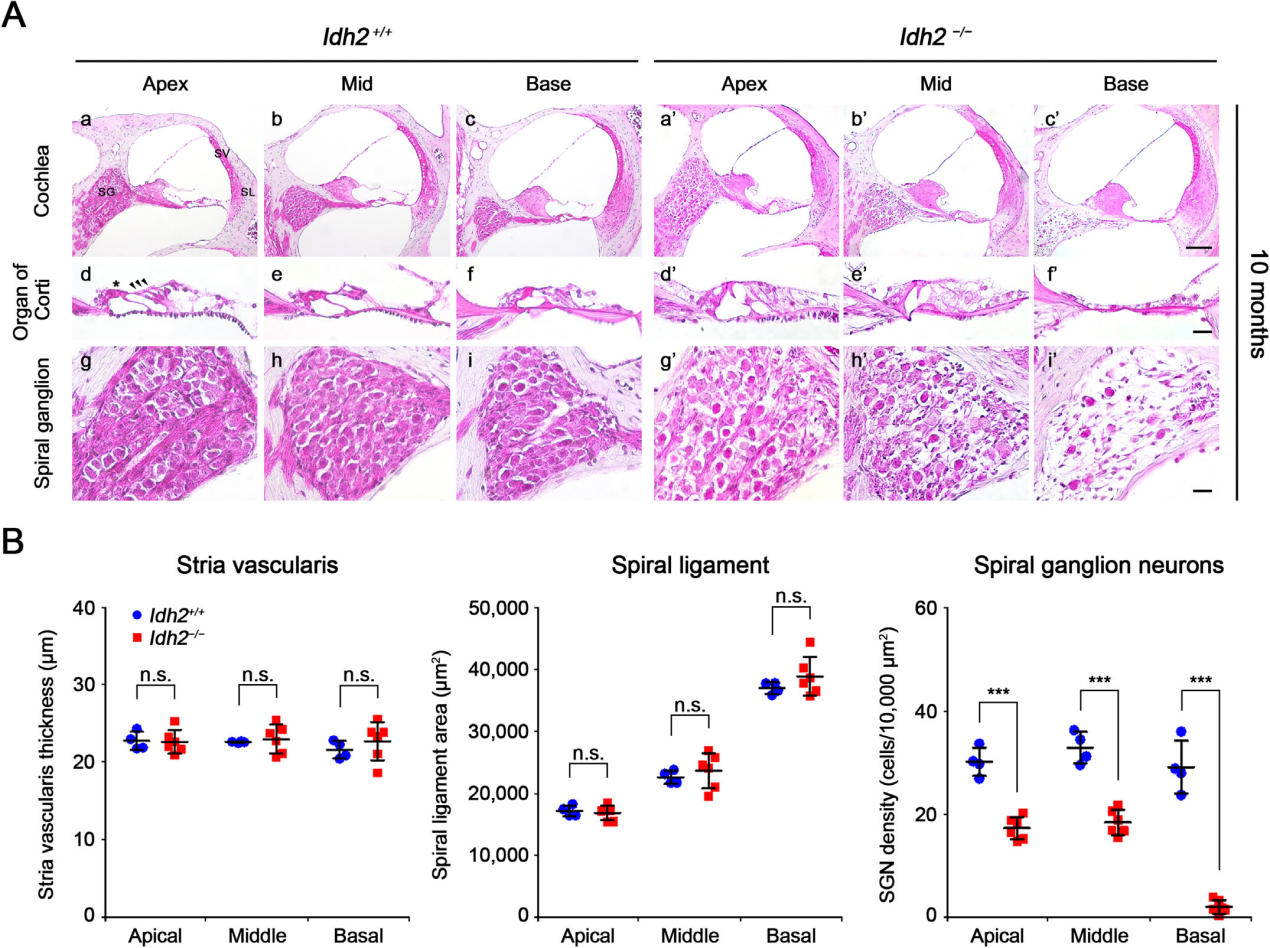
**Histological evaluation of cochlea from*****Idh2***^***+/+***^**and*****Idh2***^***−/−***^**mice.** (A) H&E staining was performed in the inner ear sections of *Idh2*^*+/+*^ (*n* = 4) and *Idh2*^*−/−*^ (*n* = 6) mice at 10 months of age. SG, spiral ganglion; SV, stria vascularis; SL, spiral ligament. Scale bars; 100 µm in (i′) and 20 µm in (c′ and f′). The arrowheads point to three rows of outer hair cells, and the asterisk (*) indicates an inner hair cell. (B) The thickness of the stria vascularis, the mean area of the spiral ligament, and the density of spiral ganglion neurons at apical, middle, and basal turns of cochlea were measured in the inner ear sections of *Idh2*^*+/+*^ (blue circles) and *Idh2*^*−/−*^ (red squares) mice at 10 months of age. ***p < 0.001; n.s., nonsignificant.

**Fig. 3:**
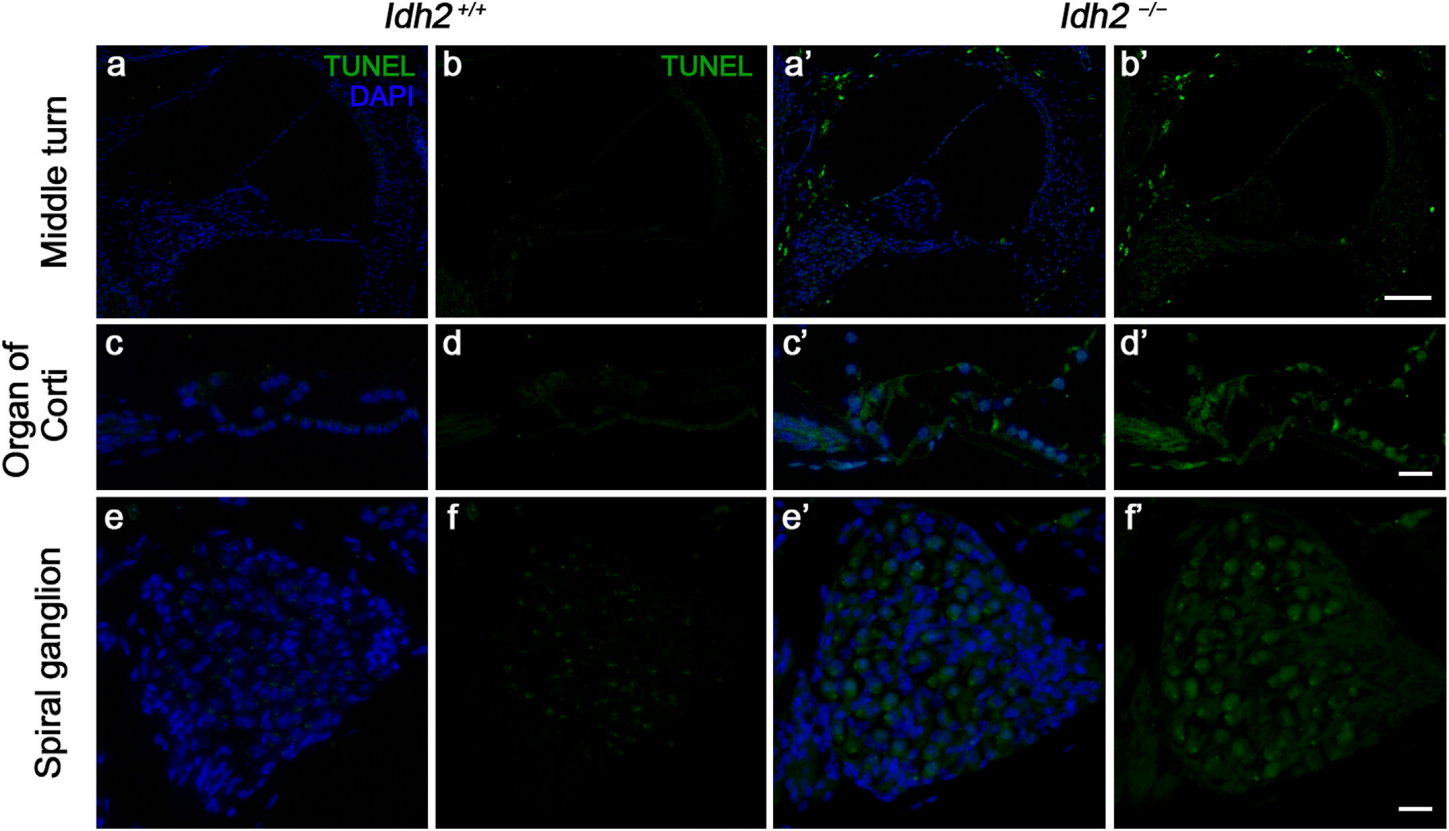
**Detection of apoptotic cell death by TUNEL assay in the cochlea from*****Idh2***^***+/+***^**and*****Idh2***^***−/−***^**mice.** Apoptotic DNA degradation was analyzed by TUNEL labeling (green) in *Idh2*^*+/+*^ and *Idh2*^*−/−*^ mice at 10 months of age, and the nuclei were counterstained with DAPI (blue). Scale bars: 100 µm in (b′) and 20 µm in (d′ and f′).

### 3.3 Loss of IDH2 function results in excessive accumulation of ROS due to decreased NADPH levels and disrupted GSH/GSSG balance in mouse cochlea

Considering the IDH2 function in mitochondria, we next investigated intracellular levels of NADPH and total ROS using immunofluorescence, IDH2 enzyme activity assay, and 3,3′-diaminobenzidine (DAB) staining in *Idh2*^*+/+*^ and *Idh2*^*−/−*^ cochlea at 10 months of age, to determine if the antioxidative function of IDH2 is indispensable for the functional maintenance and survival of hair cells and SGNs (Fig. 4). The immunofluorescence staining results indicated that the fluorescence signal for NADPH was detected throughout the entire inner ear sections in both *Idh2*^*+/+*^ and *Idh2*^*−/−*^ mice. However, the signal intensity was much weaker in *Idh2*^*−/−*^ cochlea than in *Idh2*^*+/+*^ (Fig. 4A). This result was confirmed by an *in vitro* enzymatic activity assay, which measures NADPH production by IDH2 in NADP^+^ containing inner ear lysates from *Idh2*^*+/+*^ and *Idh2*^*−/−*^ mice. The relative efficiency of NADPH production in IDH2-deficient inner ear lysate was approximately 12% compared to *Idh2*^*+/+*^ cochlea (Fig. 4B). Consequently, the intracellular level of GSH that is generated by the NADPH-driven reduction in GSSG was significantly decreased, leading to an increase in the GSSG/GSH ratio, which indicated disruption of the redox balance in the IDH2-deficient inner ear (Fig. 4C). Finally, increased intracellular ROS levels in *Idh2*^*−/−*^ cochlea, including in the organ of Corti and the spiral ganglia, was detected by DAB staining (Fig. 4D). These results demonstrated that loss of IDH2 activity decreased the levels of NADPH and GSH, leading to redox imbalance, which might cause apoptosis of hair cells and SGNs by abnormal ROS accumulation in *Idh2*^*−/−*^ mice.

**Fig. 4:**
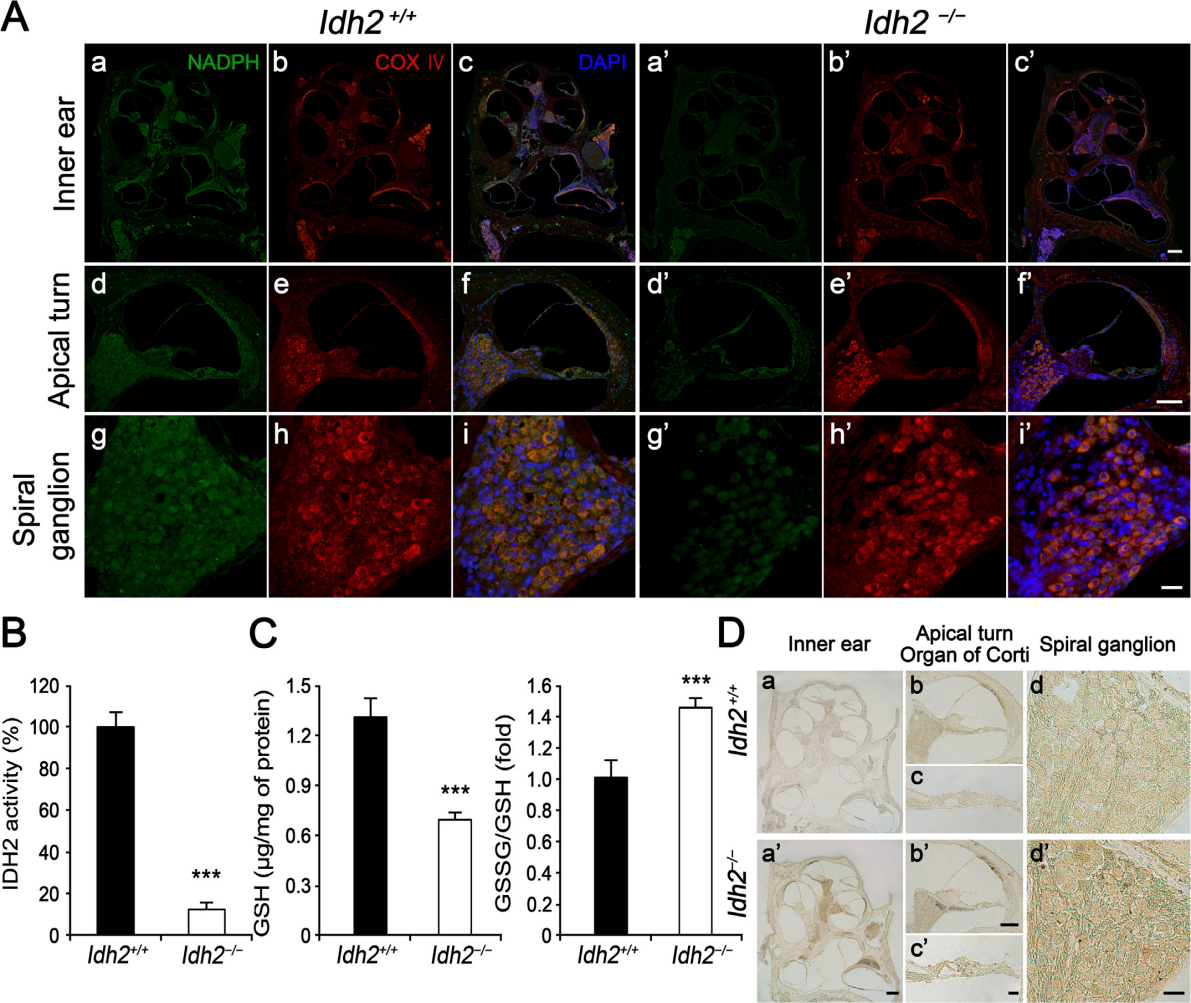
**Redox status and oxidative damage observed in the inner ear of*****Idh2***^***−/−***^**mice.** Intracellular levels of NADPH and ROS accumulation were compared between *Idh2*^*+/+*^ and *Idh2*^*−/−*^ mouse cochlea by immunofluorescence and DAB staining at 10 months of age. (A) Intracellular NADPH (green) levels in the inner ears of *Idh2*^*+/+*^ (*n* = 3) and *Idh2*^*−/−*^ (*n* = 3) mice were compared at 10 months of age. COX IV (red) was used as a mitochondrial marker [32], and the nuclei were counterstained with DAPI (blue). Scale bars: 200 µm in (c′), 100 µm in (f′) and 20 µm in (i′). (B) The enzyme activity of IDH2 that produces NADPH was measured in the inner ear whole protein fractions (*n* = 3). (C) Total GSH level and the [GSSG]/[GSH] ratio were measured (*n* = 3). The values were presented as the fold change over the levels observed in *Idh2*^*+/+*^ mice. Data are shown as the means ± SEM. **p < 0.005, *** *p* < 0.001 (D) Oxidized levels of the inner ear sections from *Idh2*^*+/+*^ (*n* = 3) and *Idh2*^*−/−*^ (*n* = 3) mice were analyzed using DAB staining. Scale bars: 200 µm in (a′), 100 µm in (b′) and 20 µm in (c′ and d′).

### 3.4 Mitochondria-targeted antioxidant MitoQ prevents H_2_O_2_-induced ototoxicity in *Idh2*^*−/−*^ cochlea

Our *in vivo* study demonstrated that the most direct cause of hearing loss observed in *Idh2*^*−/−*^ mice was the accumulation of excessive ROS, leading to apoptosis of hair cells and SGNs. Because IDH2 is a major NADPH-producing enzyme in the mitochondrial redox system and ROS-induced mitochondrial damage directly affects cell survival [1], we hypothesized that the administration of effective mitochondrial antioxidants might protect or promote the recovery of cells from ROS-induced apoptosis in *Idh2*-deficient mouse cochlea. To verify our hypothesis, we induced acute oxidative stress in *Idh2*^*+/+*^ and *Idh2*^*−/−*^ mouse cochlear explants by treatment with H_2_O_2_, leading to ROS-induced cell damage. Before the addition of H_2_O_2_, the mitochondria-targeted antioxidant, MitoQ, was pre administered to the cochlear explants to examine whether it could protect *Idh2*-deficient hair cells from H_2_O_2_ (ROS)-induced apoptosis. First, *Idh2*^*+/+*^ cochlear explants were treated with 0.025, 0.05, or 0.1 mM H_2_O_2_ for 5 days to determine the optimal toxic dose, and hair cell damage was visualized by immunostaining of cochlear whole-mount with phalloidin. Treatment with H_2_O_2_ led to the degeneration of stereocilia and the loss of hair cells in a dose-dependent manner, and the damage gradually became more severe from the apical to basal turn (Supplementary Fig. 4). Finally, a 0.05 mM concentration that contributed to obvious but mild hair cell defects in *Idh2*^*+/+*^ was selected as the H_2_O_2_ treatment dose for all subsequent experiments.

**Fig. 5:**
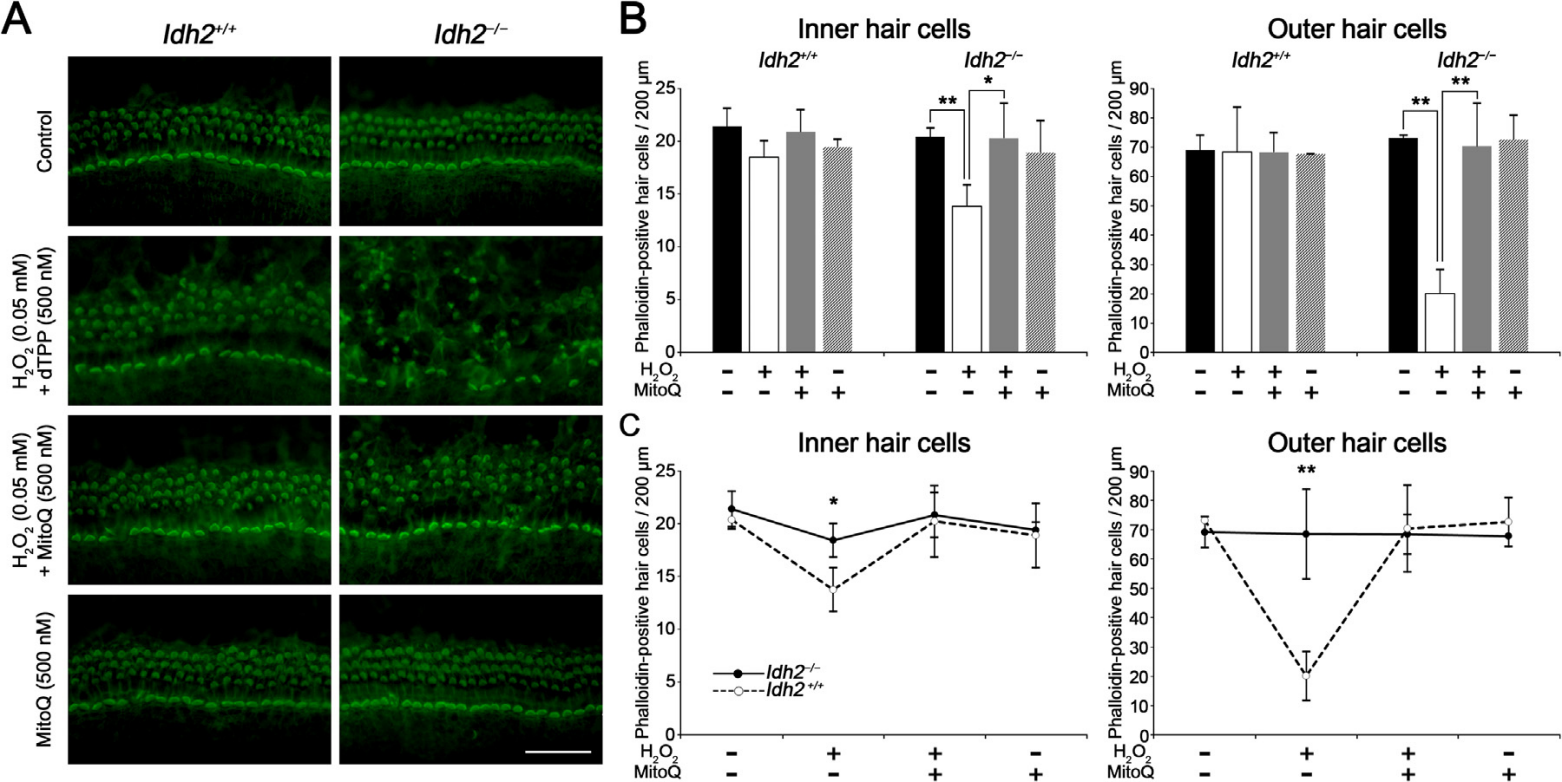
**Evaluation of protective effect of MitoQ on H**_**2**_**O**_**2**_**-induced ototoxicity in*****Idh2***^***+/+***^**and*****Idh2***^***−/−***^**cochlear explants.** (A) Immunofluorescent images represent the organ of Corti explants from each experimental group. Hair cells were stained with phalloidin (green). Scale bars: 50 µm. (B and C) Quantitative comparison of phalloidin-positive inner and outer hair cells in *Idh2*^*+/+*^ and *Idh2*^*−/−*^ mice within a 200-μm region of the organ of Corti (*n* = 3). Data are shown as the means ± SEM. *p < 0.05, ** *p* < 0.005.

The protective effect of MitoQ against H_2_O_2_-induced hair cell loss was investigated by pre-treatment with 500 nM MitoQ, followed by post-treatment with 0.05 mM H_2_O_2_ in *Idh2*^*+/+*^ and *Idh2*^*−/−*^ cochlear explants. When the cochlear explants were exposed to only H_2_O_2_ and a negative control of MitoQ, dTPP, *Idh2*-deficient cochlear explants exhibited highly severe damage in both inner and outer hair cells showing disarrangement of hair cell rows and stereocilia degeneration, while only mild hair cell migration was observed without noticeable loss of hair cells in *Idh2*^*+/+*^ cochlea (Fig. 5A). This means that loss of IDH2 function makes hair cells much susceptible to H_2_O_2_-induced oxidative stress. However, MitoQ pre-treatment before adding H_2_O_2_ remarkably reduced hair cell loss in *Idh2*^*−/−*^ cochlear explants, suggesting that the mitochondrial antioxidative effect of MitoQ protects hair cells from H_2_O_2_-induced degeneration even when the cells are deficient in IDH2. Treatment with only MitoQ showed no evident cytotoxicity. These results were quantitatively measured by counting the average number of stereocilia-positive hair cells in the 200-μm cochlear region of the middle turn. In *Idh2*^*+/+*^ cochlea, neither H_2_O_2_-induced damage nor MitoQ-dependent recovery of hair cells were significant. In contrast, in *Idh2*^*−/−*^ cochlea, hair cell loss by H_2_O_2_ treatment was highly significant and was dramatically recovered by pre-treatment with MitoQ (Fig. 5B). Importantly, although H_2_O_2_ treatment led to a significant decrease in hair cell survival in *Idh2*^*−/−*^ cochlea compared with *Idh2*^*+/+*^ cochlea (Fig. 5C), MitoQ almost completely neutralized the H_2_O_2_-induced ototoxicity, contributing to restoration of the survival rate of *Idh2*^*−/−*^ hair cells to normal levels in both inner and outer hair cells. This phenomenon was more prominent in OHCs than in IHCs. This result strongly suggests that IDH2 plays an indispensable role in removing mitochondrial ROS, which is essential for the functional maintenance and survival of hair cells. Furthermore, it suggests a strong possibility that the protective or therapeutic effect of various mitochondria-targeted antioxidant reagents could be examined to overcome mitochondrial ROS-induced ototoxicity using this model.

**Fig. 6:**
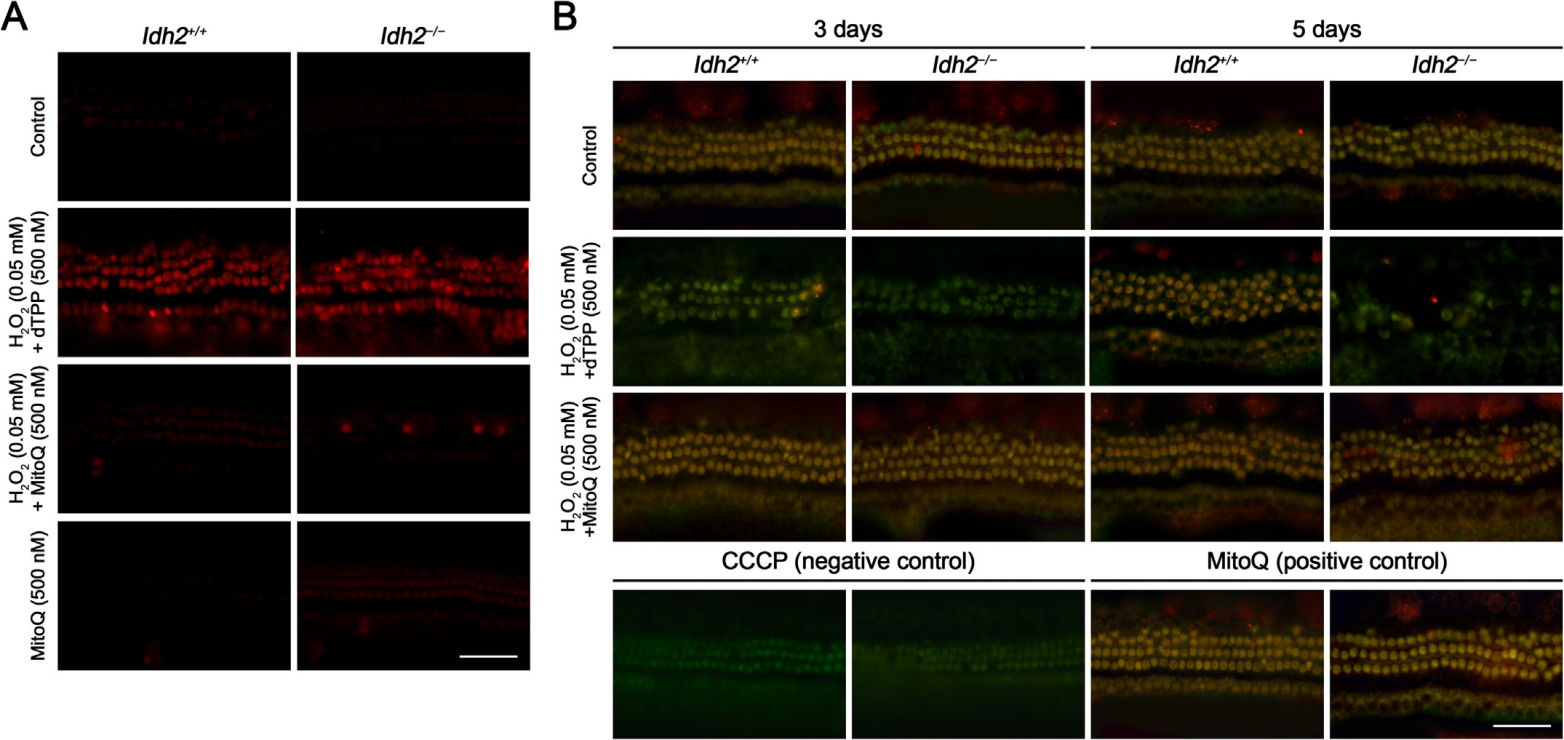
**MitoQ-mediated hair cell protection against ROS accumulation and loss of mitochondrial membrane potential caused by H**_**2**_**O**_**2**_**insults**. The organ of Corti explants of the *Idh2*^*+/+*^ and *Idh2*^*−/−*^ mice were treated with H_2_O_2_ and/or MitoQ for 3 or 5 days. (A) Level of mitochondrial ROS accumulation in the hair cells (red) was examined using MitoSOX-red staining 3 days after H_2_O_2_ treatment. Scale bars: 50 µm. (B) Mitochondrial membrane potential was tested using MitoProbe™ JC-1 assay in the *Idh2*^*+/+*^ and *Idh2*^*−/−*^ cochlea, 3 and 5 days after H_2_O_2_ treatment. Shift of the fluorescence emission from green to red were detected in the cells that are maintaining normal range of mitochondrial membrane potential. Scale bars: 50 µm.

Direct evidence of mitochondrial damage due to oxidative insults in *Idh2*^*−/−*^ mouse cochlea was confirmed by analyzing mitochondrial ROS levels and *ΔΨ*_*m*_ in cochlear explants using the mitochondrial ROS indicator MitoSOX-red and the mitochondria-accumulated fluorescent dye MitoProbe JC-1, respectively. The data showed that H_2_O_2_ stimuli led to increased mitochondrial ROS levels (Fig. 6A) and depolarization of *ΔΨ*_*m*_ (Fig. 6B) in *Idh2*^*−/−*^ mouse cochlea 3 days after H_2_O_2_ treatment, but this damage was effectively prevented by MitoQ. These results provide strong evidence that the antioxidative activity of MitoQ protects hair cells from H_2_O_2_ by reducing ROS levels and by maintaining *ΔΨ*_*m*_. Interestingly, increased ROS levels and loss of *ΔΨ*_*m*_ were also detected in *Idh2*^*+/+*^ cochlea 3 days after H_2_O_2_ treatment, indicating that an excessive amount of ROS might lead to temporary mitochondrial damage even in normal cells. However, 5 days after H_2_O_2_ treatment, *ΔΨ*_*m*_ of *Idh2*^*+/+*^ hair cells was substantially restored by the endogenous antioxidative system, while *Idh2*-deficient hair cells finally failed to survive. This means that a lack of IDH2 leads to the depolarization of *ΔΨ*_*m*_ by excessive ROS, resulting in hair cell loss caused by mitochondrial damage.

**Fig. 7:**
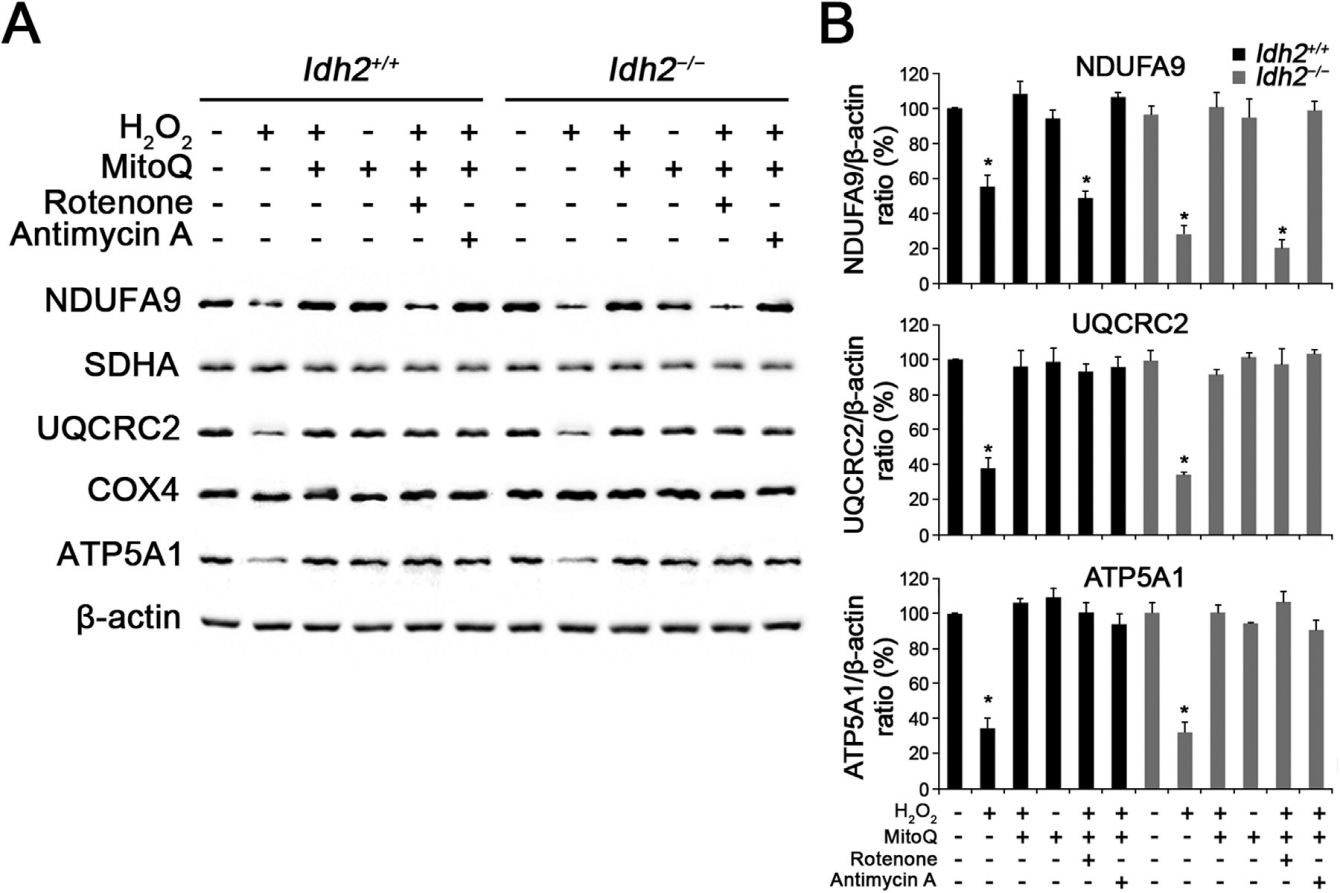
**Decreased expression levels of mitochondrial respiratory chain complexes in H**_**2**_**O**_**2**_**-treated cochlear explants.** (A) The organ of Corti explants of the *Idh2*^*+/+*^ and *Idh2*^*−/−*^ mice were treated with H_2_O_2_ and/or MitoQ for 3 days. Altered expression levels of the oxidative phosphorylation (OXPHOS) subunits I, III and V were detected in the total protein lysates from H_2_O_2_-treated cochlea by Western blot analysis. β-actin served as a loading control. (B) Protein levels of OXPHOS complexes were calculated by densitometric analysis using ImageJ software (*n* = 3) Data are shown as the means ± SEM. *p < 0.05.

**Fig. 8:**
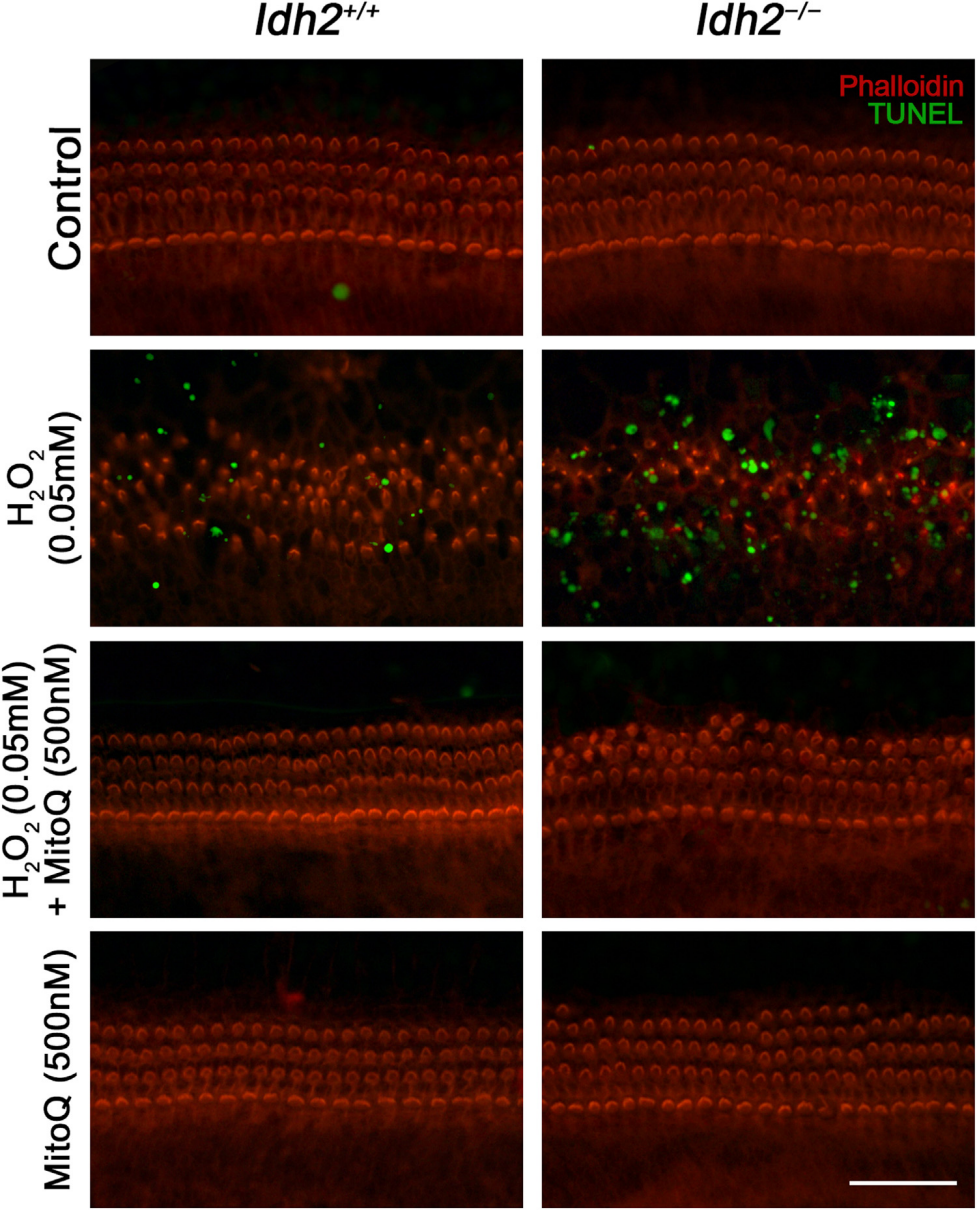
**Inhibition of apoptosis by MitoQ in H**_**2**_**O**_**2**_**-treated cochlear hair cells.** Apoptotic cell death were determined by a TUNEL assay in cochlear explants of *Idh2*^*+/+*^ and *Idh2*^*−/−*^ mice. Microscopic images represent TUNEL (green) and phalloidin (red) labeled cells. Scale bars: 50 µm.

To determine the underlying cause of depolarized *ΔΨ*_*m*_, we investigated the expression of the mitochondrial respiratory chain. Because *ΔΨ*_*m*_ is known to be generated by the mitochondrial respiratory chain composed of four enzyme complexes (complex I – IV), cytochrome c, and ATP synthase (complex V) [28], [29], changes in the expression levels of these protein complexes were examined by detecting subunits of each complex using western blot analysis in *Idh2*^*+/+*^ and *Idh2*^*−/−*^ cochlear explants, 3 days after H_2_O_2_ treatment. The results showed that subunits of three enzyme complexes, NDUFA9 (complex I), UQCRC2 (complex III), and ATP5A1 (complex V), were decreased by H_2_O_2_-induced oxidative stress, while MitoQ pre-administered hair cells maintained intact expression of these subunits at normal levels (Fig. 7). Particularly, although rotenone, a proven complex I inhibitor, decreased the NDUFA9 (complex I) expression even in the presence of MitoQ, subsequent complex subunits (II – V) were not affected by the NDUFA9 damage. In addition, complex III inhibition by treatment of antimycin A that is a complex III inhibitor, was prevented by MitoQ pre-treatment (Fig. 7A). It suggests that MitoQ might have a significant role for maintenance of normal expression and function of electron transport chain, as well as its antioxidative function. Eventually, ROS-induced mitochondrial dysfunctions triggered apoptosis, and depletion of IDH2 more strongly promoted apoptosis (Fig. 8). It suggests that the loss of *ΔΨ*_*m*_ in hair cells lacking IDH2 was due to ROS-induced degradation of mitochondrial respiratory complexes resulting in mitochondrial dysfunctions and subsequent apoptosis, and that MitoQ has a powerful protective effect against mitochondrial damage through its antioxidative activity.

Together, our *ex vivo* studies using cochlear explants demonstrated that IDH2 plays a crucial role in hair cell survival by regulating mitochondrial ROS levels and that hair cell degeneration caused by IDH2 deficiency can be surprisingly prevented by MitoQ. Therefore, *Idh2*^*−/−*^ mice could be used as a valuable animal model to evaluate the therapeutic effects of various antioxidant candidates to overcome ROS-induced hearing loss.

## 4 Discussion

In the present study, we identified that IDH2 deficiency cause progressive hearing decline in mice. Excessively accumulated mitochondrial ROS induced depolarization of the *ΔΨ*_*m*_, which resulted in mitochondrial dysfunction leading to apoptosis of hair cells and SGNs in *Idh2*^*−/−*^ mice and their cochlear explants. Thus, IDH2 is indispensable for the functional maintenance of mitochondria and survival of hair cells and for the SGNs that play critical roles in the hearing pathway. Extensive expression of IDH2 in cochlea, including hair cells and SGNs [27], and its mitochondrial-targeted intracellular localization [27] strongly support our results obtained in *Idh2*^*−/−*^ mice. In particular, the western blot analysis suggested that the most direct cause of the decreased *ΔΨ*_*m*_ observed in *Idh2*^*−/−*^ cochlea might be the loss of mitochondrial respiratory chain complexes I, III and ATP synthase (complex V). The mitochondrial respiratory chain, which consists of four enzyme complexes (I – IV) and the ATP synthase, generates *ΔΨ*_*m*_ by transferring protons from the mitochondrial matrix to the interspace between the inner and outer mitochondrial membranes, and it is also well known as a major source of superoxide (·O_2_^-^). It has been demonstrated that functional impairment in respiratory chain complexes is highly linked to mitochondria-triggered apoptosis accompanied by cytochrome c release and caspase activation [28], [29]. Inhibited complex I function led to apoptosis through caspase-3-like protease activation in ML-1a cells and cultures of dopaminergic cells [30], [31], and respiratory chain dysfunctions induced by pathogenic gene mutations altered the level of caspase-3 activation in response to mitochondrial stress-mediated apoptotic stimuli [32]. Interestingly, each enzyme complex has differential sensitivity to endogenous oxidative stress, and their levels were restored by the administration of specific antioxidants. In optic nerve head astrocytes, H_2_O_2_-induced oxidative stress significantly increased the only complexes I, II and IV, and pre-treatment with coenzyme Q_10_ preserved their expression levels at a normal state [33]. Another *in vivo* study using SOD2 null mice revealed that complexes I – IV, but not V, were sensitive to mitochondrial ROS in *Sod2*^*−/−*^ mice [34]. Generally, protein oxidation by ROS occurs at particular amino acid residues of a certain protein, rather than at random. Moreover, transition metals bound to proteins are known as the strongest target of initial oxidation, reacting with ROS and form hydroxyl radicals (·OH) [35]. Our data suggest that only mitochondrial respiratory chain complexes I, III and ATP synthase were particularly sensitive to oxidative insult by H_2_O_2_ in hair cells lacking IDH2, which finally resulted in apoptosis. Complex I, II and III commonly have transition metal cofactors (iron-sulfur clusters) that are sensitive to mitochondrial ROS, and they are functionally associated with each other [36]. Complex I and III share important physiological characteristics. As the major contributors of mitochondrial ROS generation,·O_2_^-^ produced from complexes I and III is converted to H_2_O_2_, which mediates intracellular signaling under normal physiological conditions, while·O_2_^-^ from complex II has been proposed to be used to open mitochondrial ATP-sensitive potassium channels [37]. Given the functional significance of respiratory complexes I, III and ATP synthase, which are responsible for the majority of ROS and ATP synthesis, respectively, our result that consistent downregulation of these complexes was confirmed by repeating three independent experiments strongly suggests that IDH2 plays a critical role in maintaining mitochondrial functions and cell survival by protecting the mitochondria from the excessive ROS that damages specific respiratory chain complexes.

The pattern of respiratory complex degeneration and loss of *ΔΨ*_*m*_ induced by H_2_O_2_ stimuli were not IDH2-deficient cell-specific defects. Three days after H_2_O_2_ treatment, ROS-induced mitochondrial damage did not significantly differ between *Idh2*^*+/+*^ hair cells and *Idh2*^*−/−*^ hair cells. Nevertheless, the intact function of IDH2 induced the hair cells to restore the mitochondrial redox balance to overcome the damage, while a lack of IDH2 eventually caused apoptotic cell death due to failure to remove mitochondrial H_2_O_2_. However, this ROS-induced mitochondrial damage leading to apoptosis was dramatically preserved by MitoQ in *Idh2*^*−/−*^ cochlea. MitoQ, an analog of ubiquinone, is a respiratory chain component and a highly effective antioxidant that prevents lipid peroxidation in mitochondria [38], [39]. By covalent conjugation of a lipophilic triphenylphosphonium cation to ubiquinone, MitoQ can pass through the phospholipid bilayers and largely accumulate into the mitochondrial inner membrane by the *ΔΨ*_*m*_ [38]. Thus, MitoQ has been widely used as an antioxidant that targets mitochondria [38], [40]. MitoQ is known to inhibit the final step of lipid peroxidation by blocking ·OH assault and is continuously recycled by returning to the active ubiquinol form by mitochondrial respiratory chain complex II [40]. As a nontoxic and effective mitochondria-targeted antioxidant, the protective effect of MitoQ on lipid peroxidation and mitochondrial damage has been determined in various studies of ROS-related diseases, including renal and liver dysfunction in sepsis, cardiac hypertrophy, and neurodegenerative disease [41], [42], [43]. Moreover, two previous *in vivo* studies found protective effects of MitoQ in the inner ear. Using gentamicin- or amikacin-treated guinea pigs, it was shown that the administration of MitoQ attenuated aminoglycoside-induced ototoxicity, leading to cochlear damage and hypoacusia [44], [45]. Although the protective effect of MitoQ against ototoxicity was determined in previous studies, an important significance of this study is the suggestion of the possibility to reverse genetic hearing loss. By providing experimental evidence of the protective potential of an antioxidant to inhibit ROS-induced cochlear damage in a transgenic mouse model, we are the first to suggest the possibility that continuous application of nontoxic supplemental agents, such as promising antioxidants, might be effective in controlling the symptoms of genetic hearing loss caused by a lack of genes involved in the cellular redox system, although not a fundamental and direct genetic correction such as virus-mediated gene transfer. In addition, although all the experiment for protective effect of MitoQ was verified using *ex vivo* organotypic culture system of cochlea, not *in vivo*, cochlea is highly distinctive organ that has structural and physiological complexity, which brings diverse limits in functional investigations *in vivo*. In that respect, organotypic culture of cochlear explants have strong advantages to explore the underlying mechanisms for a particular pathogenesis or development, because the cultured cochlear explants have been proven to maintain intact conformation of the hair cells and neuronal innervation with following normal processes of development during it is cultured [46], [47]. Importantly, most of hearing loss studies that performed both *ex vivo* and *in vivo* experiments have shown strong consistency in the results between these two systems [48], [49], [50], [51], which suggest that *ex vivo* studies also could provide significant and reliable massages.

Thus far, aging has been considered as the process of ROS-induced damage accumulation [52], which causes mitochondrial dysfunction resulting in age-related disorders [53]. Hearing loss caused by constant accumulation of ROS in inner ear cells is also known to be a consequence of aging; thus, the genes contributing to intracellular redox balance have been concatenated with ARHL, *i.e.,* presbycusis [54]. IDH2, which converts NADP^+^ to NADPH to maintain the GSH/GSSH ratio, has been highlighted as the major contributor to the antioxidant defense system in various tissues or cells [15], [16], [21], and several *in vivo* studies found that IDH2 deficiency causes organ dysfunctions by mitochondrial damage with aging in old mice [23], [55], [56]. A recent study performed by White *et al.* demonstrated that lack of IDH2 resulted in apoptosis of hair cells and SGNs due to accumulation of mitochondrial oxidative stress, which accelerates ARHL in CBA/CaJ male mice. They suggested that decreased NADPH redox status caused by IDH2 deficiency disables thioredoxin pathway particularly, rather than glutathione pathway in mitochondria, eventually leading damages of hair cells and SGNs [56]. This result showed two major differences with our study, the onset age of hearing loss and its major underlying mechanism. First in this study, a significant shift in the ABR threshold was found after 3 months of age in *Idh2*^*−/−*^ mice, which was much earlier than the general onset age of ARHL in mice [57]. A possible explanation can be found in the genetic background of the C57BL/6 mouse strain of the *Idh2*^*−/−*^ mice used in this study. The C57BL/6 mouse strain is known as homozygous for a specific mutation in the *Cdh23* gene (c.753A>G). Because *Cdh23* encodes a component of the tip-link that connects the stereocilia of hair cells, dysfunctional CDH23 generated by the c.753A>G mutation makes the mice susceptible to ARHL [57], [58]. The C57BL/6 strain, which exhibits a critical pattern of ARHL by 12–15months of age, has been used for various studies associated with aging [59], [60]. This genetic background might accelerate the onset age of hearing loss caused by IDH2 deficiency. Although this strain essentially has ARHL even in wild type, the *Idh2*^*−/−*^ mice exhibited obvious and significantly greater hearing deterioration and histological defects than the *Idh2*^*+/+*^ mice. Moreover, only *Idh2*^*+/+*^ and *Idh2*^*−/−*^ mouse littermates were used in all experiments to minimize individual differences and variations in the maternal environment. Therefore, we conclude that our data are sufficient to provide evidence that the obviously defective phenotype found in *Idh2*^*−/−*^ mice and the disrupted redox balance observed in IDH2-deficient cochlear explants were caused by the loss of IDH2 function. Second, our study verified that IDH2 deficiency lead to an increase in the GSSG/GSH ratio leading mitochondrial damage, suggesting that mitochondrial glutathione pathway is significantly regulated by IDH2. This result was highly consistent with other IDH2 studies. In several organs, the role of IDH2 has been determined to be essential for maintenance of mitochondrial glutathione status to prevent diseases including cardiac hypertrophy [18], and renal dysfunction [20], [61]. Considering that IDH2 is an upstream regulator of the glutathione-dependent antioxidant defense pathway in mitochondria, it could be expected that IDH2 function might influence the overall mitochondrial redox balance. Thus, using this *Idh2*^*−/−*^ mouse model for functional studies of ROS-related hearing loss will allow us to extend the range of applicable antioxidants. This means that various types of mitochondria-targeted antioxidant that interventions at different stages of the ROS cascade can be evaluated for their therapeutic effect using this valuable *Idh2*^*−/−*^ mouse model.

In summary, our *in vivo* and *ex vivo* studies using *Idh2*^*−/−*^ mice demonstrated that IDH2 plays a crucial role in the survival of hair cells and SGNs by regulating mitochondrial ROS levels, which proves the powerful impact of IDH2 in the functional maintenance of cochlear cells. Degeneration of the cells caused by IDH2 deficiency can be prevented by MitoQ, suggesting a possibility that genetic hearing loss caused by functional loss of ROS-related genes might be prevented by nongenetic agents. Finally, this study suggests that this *Idh2*^*−/−*^ mouse model might be of great value in the development of novel therapeutic agents to overcome mitochondrial ROS-induced hearing loss in humans.

## Figures and Tables

**Fig. 1:**
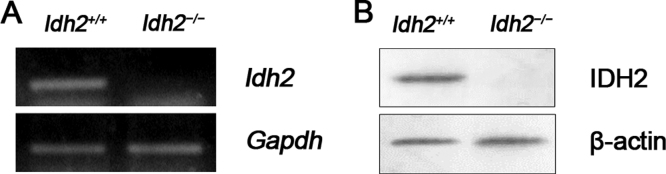
**Analysis of IDH2 expression in the inner ear of*****Idh2***^***+/+***^**and*****Idh2***^***−/−***^**mice.** (A) Loss of Idh2 mRNA expression in the inner ear of *Idh2*^*−/−*^ mice at P20 was confirmed by RT-PCR analysis. *Gapdh* was used as a loading control. (B) Lack of IDH2 protein in the inner ear of *Idh2*^*−/−*^ mice at P20 was confirmed by western blot analysis using an anti-IDH2 antibody. β-actin was used as a loading control.

**Fig. 2:**
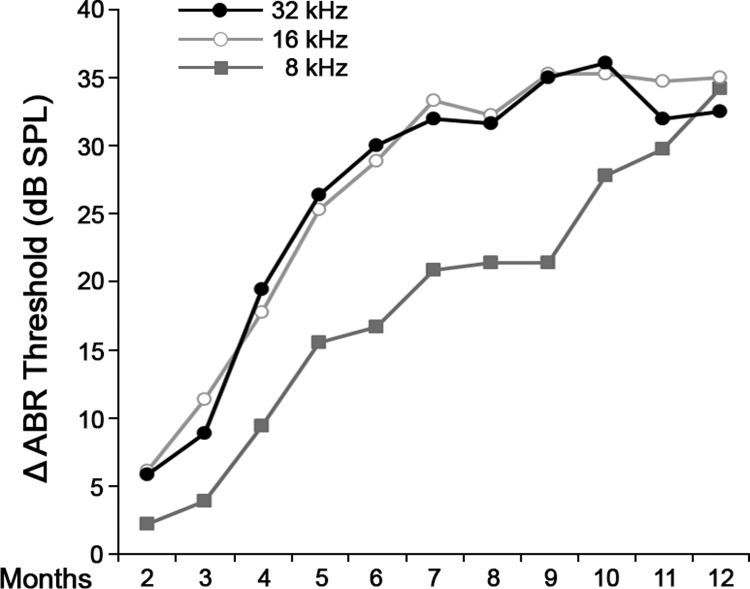
**Relative comparison of hearing loss progression at each frequency.** Differences in the mean ABR threshold between *Idh2*^*+/+*^ and *Idh2*^*−/−*^ mice are shown in the graph for 8 (squares), 16 (white circles) and 32 kHz (black circles).

**Fig. 3:**
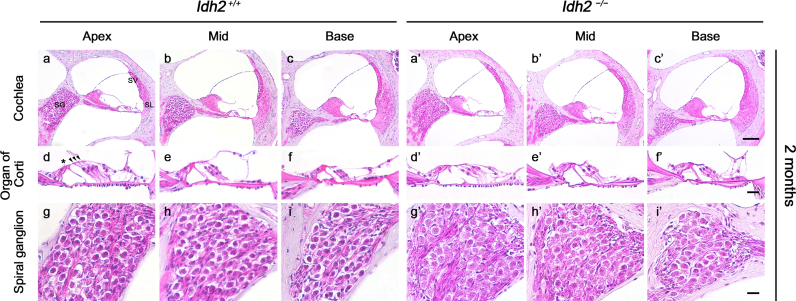
**Histological evaluation of cochlea from*****Idh2***^***+/+***^**and*****Idh2***^***−/−***^**mice.** H&E staining was performed in the inner ear sections of *Idh2*^*+/+*^ and *Idh2*^*−/−*^ mice at 2 months of age. SG, spiral ganglion; SV, stria vascularis; SL, spiral ligament. Scale bars, 100 µm in (i′) and 20 µm in (c′ and f′). The arrowheads point to three rows of outer hair cells, and the asterisk (*) indicates an inner hair cell.

**Fig. 4:**
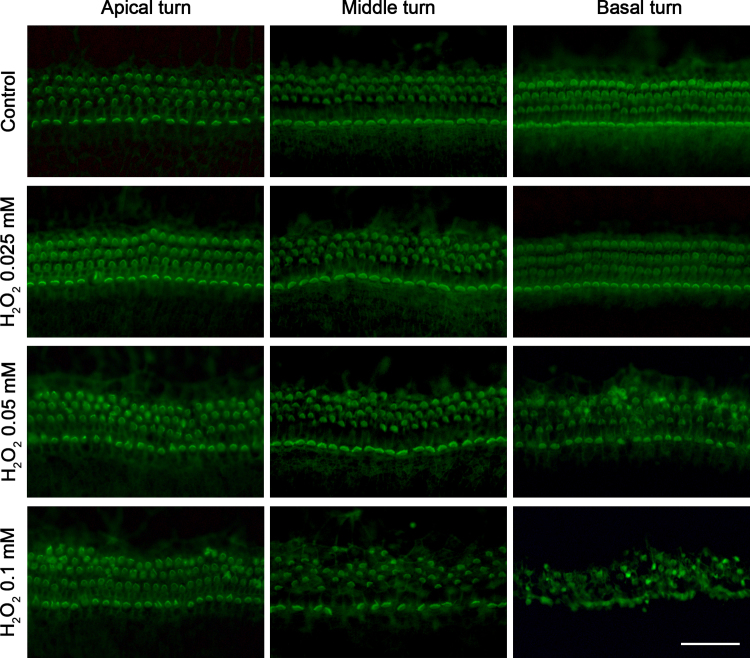
**Hydrogen peroxide-induced oxidative damage to hair cells in the cochlear explants from*****Idh2***^***+/+***^**mice.** The organs of Corti explants of newborn *Idh2*^*+/+*^ mice were treated with the indicated concentrations of hydrogen peroxide for 5 days. The cochlear explants were stained with phalloidin to label the outline of the hair cells. Scale bars: 50 µm.
